# A survey of downstream applications of evolutionary scale modeling protein language models

**DOI:** 10.1002/qub2.70013

**Published:** 2025-09-21

**Authors:** Qingyu Yang, Jiale Yu, Jie Zheng

**Affiliations:** ^1^ School of Information Science and Technology ShanghaiTech University Shanghai China; ^2^ Shanghai Engineering Research Center of Intelligent Vision and Imaging Shanghai China

**Keywords:** BERT, fine‐tuning, pretraining, prompting, protein design, protein function, protein language model, Transformer

## Abstract

The evolutionary scale modeling (ESM) series is promising to revolutionize protein science and engineering through large language models (LLMs), providing a robust framework for understanding the relationships among sequences, structures, and functions of proteins. Trained on a large number of unlabeled protein sequences, ESM models are able to capture intricate patterns of mutation and conservation, yielding insights into the structural and functional properties of proteins. Despite a growing body of literature surrounding ESM, existing surveys often fail to comprehensively describe its advancements or applications in a focused manner. This survey covers the latest developments of ESM, categorizing them into techniques of using ESM and downstream applications. Approximately 100 papers are selected and analyzed, highlighting recognized and innovative studies that exemplify the impact of ESM. Furthermore, we critically discuss the strengths and limitations of ESM to envision future applications. This review provides a valuable resource for researchers seeking to explore the power of ESM models and the emerging applications of LLMs in biology and medicine.

## 1 INTRODUCTION

The advent of the evolutionary scale modeling (ESM) series of protein language models (PLMs) [1, 2, 3, 4, 5, 6] is a significant innovation in the convergence of large language models (LLMs) with protein representation. These models, trained on large amounts of unlabeled protein sequence data, distill the intricate patterns of mutation and conservation that have sculpted protein families through evolutionary history. ESM models are significant primarily because of their ability to model proteins and mirror biological evolution. It provides a foundation upon which many downstream applications can be constructed, offering insights into various aspects of proteins.

Given the rapid development of ESM and its applications in biotechnology and medicine, a survey of the latest advances is both timely and necessary. Although there are already some reviews mentioning ESM [7, 8, 9, 10, 11], they either focus on specific subdomains of proteins rather than LLMs [8, 9] or lump together many language models and ignore the specificity of ESM [7, 10, 11]. In addition, the ESM series has been expanded and refined over time, but few reviews have a comprehensive account of them. Hence, this review aims to describe the latest technical innovations around ESM with a focus on its downstream applications.

First, we provide a brief overview of the ESM series. Next, we classify the downstream applications of ESM from two perspectives: technical approaches and application domains. Finally, we discuss the strengths and limitations of ESM, as well as its potential future applications. For a comprehensive survey, we selected approximately one hundred representative papers from all papers that have cited at least one of the six original publications of ESM. All of these selected works have been cited at least five times. Given the high volume of citations and the diversity of downstream research associated with ESM, we may not capture all valuable studies in this survey. However, we believe that our review covers most major research directions and the most influential works. Readers are encouraged to explore additional studies following the framework provided here.

## 2 OVERVIEW OF ESM SERIES

**FIGURE 1:**
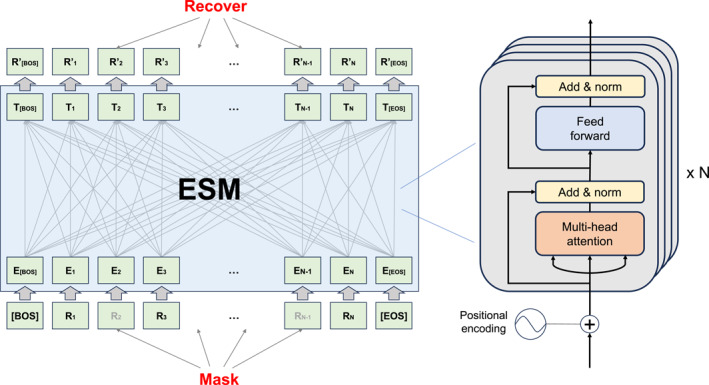
The architecture of evolutionary scale modeling (ESM) models.

The earliest and foundational work of the ESM series is ESM‐1b [1], a general‐purpose PLM. ESM‐1b adopts a BERT‐style [12] Transformer [13] architecture (see Figure 1) and is trained using the masked language modeling (MLM) [1] objective on 250 million protein sequences from UniParc [14]. MLM means that for each sequence *x*, ESM randomly samples a set of positions *M* to mask, that is, replacing the true token of amino acid at each index *i* with the mask token. For each masked token, it independently minimizes the negative log‐likelihood of the true amino acid *x*_*i*_ given the masked sequence *x/M* as context, as shown in Equation (1). The model not only validated the effectiveness of scaling laws in PLMs but also achieved state‐of‐the‐art (SOTA) performance on several downstream tasks at the time of its release.

(1) $$L_{\text{MLM}}=E_{x∼X}E_{M}\sum_{i\in M}-\log\;p\left(x_{i}|x_{M}\right).$$

Subsequent variants of ESM were built on ESM‐1b by extending it across various protein tasks. Multiple sequence alignment (MSA) Transformer (ESM‐MSA‐1b) [2] replaces common attention with tied row attention and untied column attention to extract and fuse embeddings from MSAs [15], enabling inference of protein structures, including contact and secondary structures. ESM‐1v [3] has the same architecture as ESM‐1b and is specialized for variant effect prediction (VEP) by being trained to score the effect of sequence mutations on protein function. ESM‐IF1 [4] connects Transformer [13] blocks after GVP‐GNN [16], a graph neural network with layers operating on collections of Euclidean vectors, and scales both of them. It can be used for fixed‐backbone sequence design (inverse folding) or predicting functional effects of protein sequence variation for given structures.

**FIGURE 2:**
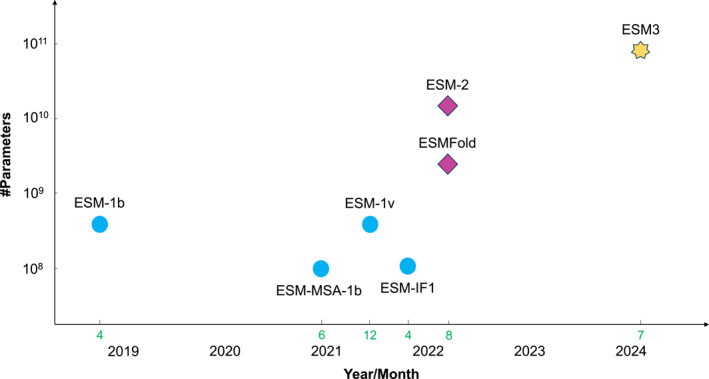
The timeline and sizes of evolutionary scale modeling (ESM) models. Different shapes and colors represent different generations. We follow the time of release as stated in ESM’s GitHub repository. Note that the paper of ESM‐1 was first released in April 2019, but the release of the final ESM‐1b model was in December 2020. For ESM‐2, we show its largest model.

ESM‐2 [5] further scales ESM‐1b and is a SOTA general‐purpose PLM. It can be used to predict a wide range of protein properties directly from individual sequences and is one of the most widely used foundation models in the field of protein science nowadays. Released in the same paper is ESMFold [5], an end‐to‐end single‐sequence 3D structure predictor that can achieve comparable performance with AlphaFold2 [17] in some important scenes. ESM3 [6] turns to protein design. It is able to jointly reason across sequence, structure, and function. The timeline and sizes of all the ESM models, as well as other information, are shown in Figure 2 and Table 1, respectively.

**TABLE 1:** Main evolutionary scale modeling (ESM) models.

Model	Main applications	Input form	Output form	Training data	# Parameters
ESM‐1b	General purpose	Protein sequence	Sequence likelihood/embedding	UniRef50 [14]	43M–670M
ESM‐MSA‐1b	Secondary structure inference	MSA	Sequence likelihood/embedding	UniRef50 [14] + MSA	100M
ESM‐1v	Variant effect prediction	Protein sequence	Sequence likelihood/embedding	UniRef90 [14]	650M
ESM‐IF1	Inverse folding, functional effect prediction of sequence variation for given structures	Protein structure	Sequence likelihood	CATH 4.3 [18] + predicted structures for UniRef50 [14]	124M
ESM‐2	General purpose	Protein sequence	Sequence likelihood/embedding	UniRef50 [14]	8M–15B
ESMFold	3D structure prediction	Protein sequence	Protein structure	PDB [19] + UniRef50 [14]	3.69B
ESM3	Protein design	Protein sequence, structure tokens, and functional tokens	Likelihood of sequence, structure, and functions	UniRef [14] + MGnify90 [20] + JGI [21] + OAS [22]	1.4B–98B

*Note*: There are also ESM‐1 series, which is the former version of ESM‐1b, and ESM‐MSA‐1, which is the former version of ESM‐MSA‐1b, but now they are rarely mentioned, so we use “1b” to represent both of them. ESM‐MSA‐1b is also called MSA Transformer. ESM‐IF1 is also called GVP‐Transformer. “Embedding” in the column of “Output form” in the table means that users often extract the output of the last layer of the model, instead of the log‐likelihood from the final projection. For “Training data,” we just list the sources of pretraining data for each model, but the versions of data and the preprocessing of data differ. Article links, codes, and other details can be seen on GitHub websites (facebookresearch/esm and evolutionaryscale/esm).

There are many PLMs [11, 23, 24, 25, 26, 27], among which ESM models are outstanding. The advantages of ESM models over other PLMs probably lie in the following: (1) currently, they perform best or close to the best in most scenarios [28, 29, 30, 31, 32, 33]; (2) they are well developed and form a complete series, with different models that can be utilized in various fields of protein research; and (3) they are widely used as base models in other works [34, 35, 36].

## 3 TECHNIQUES OF USING ESM

**FIGURE 3:**
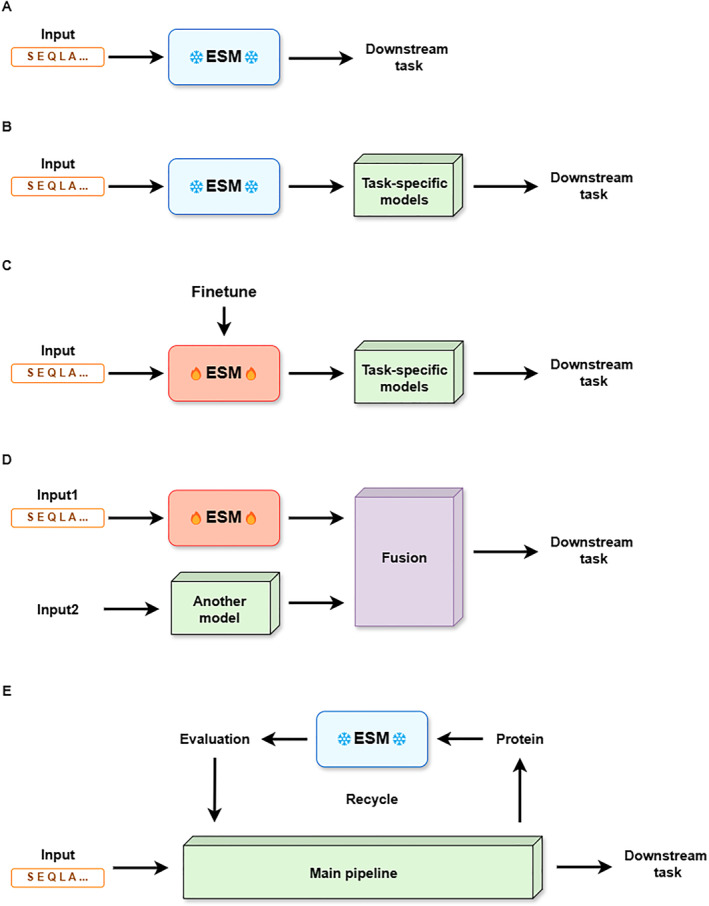
The techniques of using evolutionary scale modeling (ESM). (A) Directly using ESM models specific for certain tasks (i.e., ESM‐IF1 and ESMFold). (B) Using ESM to learn protein representations and integrating with other models. (C) Fine‐tuning ESM instead of directly using its embeddings. (D) To be fused with other models’ outputs for multimodality. (E) Evaluation or validation of other models.

The representation learning capabilities of ESM serve as the basis of various downstream tasks. ESM’s output in the form of protein’s representation acts as the conduit between raw sequence data and high‐level features that machine learning (ML) models can exploit. This is referred to as the “pretraining‐fine‐tuning” paradigm in the field of language models. As shown in Figure 3, there are different techniques of using ESM, as elaborated in the following subsections.

### 3.1 Direct use

“Direct use” means that for models that have already performed specific functions and output in a format suitable for a certain task, they can be directly used for various subtasks in that field. ESM‐IF1 is constantly used for fixed‐backbone protein design. ESMFold, as one of the SOTA protein structure prediction methods, is frequently utilized to predict protein structures in various scenes or compared with other structure prediction methods such as AlphaFold2. Several studies [37, 38, 39] have shown that ESMFold is much faster than AlphaFold2 with minor accuracy loss. De novo and orphan proteins are the focal experimental subjects of these studies and are also areas where ESMFold excels in prediction [39, 40].

### 3.2 Integration with task‐specific models

In addition to direct applications, there are integrations of ESM with other models. PLMs such as ESM‐1b and ESM‐2 are followed by task‐specific models, and conducting supervised training on the models solves various downstream prediction tasks. In such works, the output from ESM is treated as a protein representation, encapsulating information about a specific modality of the protein, and is utilized in conjunction with various other models to accomplish a wide range of downstream tasks.

ESM can be followed by an multi‐layer perceptron (MLP) layer (i.e., head/classifier/predictor) for various downstream tasks [41, 42]. This approach is mainly used in benchmarking studies, where, although ESM models show superior performance, they are not the best models in all cases [28, 29, 30, 31, 32, 33, 43, 44]. Among several well‐known PLMs, ESM and ProtT5 [24] show the best performance in different scenarios and on different metrics (with ProtT5 slightly better than ESM overall) [29, 43, 44, 45], whereas ProteinBERT [23] tends to perform slightly poorer [28, 43]. For example, in the “MCC per location” results on the Swiss‐Prot [14] cross‐validation dataset in Ref. [45], ESM‐1b reaches the highest score in “extracellular” and “golgi apparatus” tasks, whereas ProtT5 performs the best in all the other tasks. However, due to the lack of interpretability of current models and the different natures of the tasks, researchers are recommended to first test and compare these models before choosing the most appropriate one when using these PLMs in real‐world scenarios.

The output of ESM can also be combined with ML techniques. Here, various ML models are often employed as classifiers [46, 47, 48, 49, 50, 51, 52], regression models [53, 54, 55], or visualization tools [1, 41, 56]. In addition, ESM models are integrated with deep learning networks. In this context, the representation from ESM serves as an input, and subsequent neural networks play crucial roles in prediction for downstream tasks. Specifically, long short‐term memory network (LSTM) [57, 58], convolutional neural network (CNN) [55, 57, 58, 59], attention mechanism [45, 56, 60], and gated recurrent unit (GRU) [61] are used as predictors of various downstream tasks. NERE [62] and DSMBind [63] both use an equivariant rotation prediction network derived from Euler’s rotation equations, which receives the embeddings of ESM‐2 as input and outputs the protein’s energy function for binding affinity prediction. Another significant work is ESMFlow [64], which combines ESMFold with flow matching [65], a generative modeling approach to learning and sampling protein conformational landscapes.

In short, ESM contains rich prior knowledge about proteins through pretraining, allowing for convenient extraction of high‐quality protein embeddings.

### 3.3 Fine‐tuning

The third major category of methods involves fine‐tuning of ESM models. Compared to works in Section 3.2, some parameters of the model are not frozen in order to explore a larger parameter space and to achieve better performance in downstream tasks.

In addition to simply adding a naive decoder/predictor (such as a fully connected layer or MLP) [28, 66, 67, 68, 69, 70], with the development of LLM technology, parameter‐efficient fine‐tuning (PEFT) has become the mainstream fine‐tuning approach because of its computational efficiency [71, 72], especially for foundation models such as ESM‐2‐650M, which has a large number of parameters. Among PEFT methods on ESM, adapter tuning [73, 74], prompt tuning [75], and LoRA [76] are among the most commonly used and effective methods [68, 77]. Adapter tuning means the incorporation of adapter modules with a bottleneck architecture within the Transformer layer of the ESM‐2 model [77, 78]. Prompt tuning generally means adding some trainable prompt before input embedding [75]. It is used in ESM‐related works to concatenate new information before a raw protein sequence [79, 80]. For example, Chen et al. [80] employed a novel masking strategy, integrating the target protein sequence with the masked ligand region as input during the fine‐tuning phase. In the generation phase, the model can accept both the target protein sequence and masking labels to facilitate the creation of peptide segments of specified lengths. Low‐rank adaptation (LoRA) [76], by introducing trainable rank decomposition matrices into the Transformer architecture, can be seen as the most widely used fine‐tuning method [68, 77, 81]. PEFT‐SP [77] is a comprehensive work comparing conditional random field, adapter tuning, prompt tuning, and LoRA on ESM‐2 for the classification of signal peptide, and it was shown that LoRA performed the best. Other fine‐tuning strategies include FusOn‐pLM [82], which unfreezes the query weights in the last 11 layers of ESM‐2 and incorporates a novel probabilistic masking strategy. AntiFold [83] employs hierarchical learning rate decay, various masking schemes, and adding Gaussian noise to the predicted structures to fine‐tune ESM‐IF1.

### 3.4 Multimodality

The embedding from a single ESM model contains information from only one modality, leading to suboptimal performance in downstream tasks that emphasize other modalities. For example, although PLMs such as ESM‐1b and ESM‐2 exhibit certain emergent capabilities after pretraining on vast amounts of unlabeled sequences, which allows them to learn some information beyond the sequences themselves, they lack explicit consideration of structural information [34], and therefore, their embeddings do not perform well in downstream tasks that focus on structural information [35]. Consequently, researchers have drawn upon the idea of information fusion, combining features from different sources, which complies with the concept of multimodality. This integration enables a more comprehensive representation of proteins by fusing information from various modalities, such as sequence and structure, thereby enhancing performance in a wide range of downstream tasks. There are mainly 3 kinds of modality fusion techniques as stated in ESM‐GearNet [35]: (1) Sequence representations are used as residue features in structure encoders; that is, ESM’s embeddings are used as (one of) the inputs or the node features of GNN, which is the most widely used approach to integrating protein sequence features and structure features [84, 85, 86, 87, 88]; (2) concatenation of sequence and structure representations [89, 90, 91, 92]; (3) sequence and other representations are combined via attention (mainly cross attention) [93, 94]. On the other hand, ESMFold can be used to provide structural information, and some works use it as input for GNNs. For example, DeepProSite [95] and GPSFun [96] input protein sequences into ESMFold and another PLM to obtain edge features and node features, respectively, thereby constructing a graph, which is subsequently used for learning through GNNs.

In multimodal information fusion, contrastive learning is an efficient strategy, which learns by maximizing the similarity between related samples while minimizing the similarity between unrelated samples [97, 98]. For example, ConPLex [99] predicts interaction and performs contrastive learning based on the cosine distance between the projection of PLM’s embedding and the drug’s Morgan fingerprint in the latent space. In their contrastive epoch, they minimize the target‐drug distance while maximizing the target‐decoy distance. One of the popular techniques of contrastive learning is CLIP [100], which jointly trains an image encoder and a text encoder to predict the correct pairings of a batch of (image, text) training examples by computing pairwise cosine similarities between the two modalities. CLIP is a widely used approach in biomedical research [101]. In protein science, PepPrCLIP [102] and Cut&CLIP [103] do CLIP on protein encoders and peptide encoders, which receive ESM embeddings as inputs. CSSP [104] is an approach that updates a sequence encoder using a pretrained structure encoder using CLIP.

Another strategy of modality fusion is based on tasks [81, 105, 106]. Training on certain auxiliary tasks before downstream prediction helps models learn other types of information. ESM‐S [105] infuses structural information into the pretrained ESM‐2 by continual training through remote homology detection before fine‐tuning on downstream tasks. FSFP [81] builds auxiliary tasks for meta‐learning, meta‐trains PLMs on the auxiliary tasks, and transfers PLMs to the target task.

Purely using embeddings from pretrained models or fine‐tuning the models is still unsatisfactory sometimes. Researchers have tried retraining ESM for performance improvement and modality compression. This requires much computational resources, though. Here, a well‐known method is SaProt [34], which first uses Foldseek [107] to represent local structures as English letters called 3Di, analogous to using 20 letters to represent amino acids, and then combines the 3Di with amino acid letters to form a “structure‐aware” protein sequence as input to ESM‐2. This is an innovative but straightforward way to fuse structural information into a sequence encoder. Other researchers consider injecting a structure encoder block into the ESM architecture [108, 109] for retraining, an idea similar to adapter tuning mentioned earlier.

### 3.5 The use of attention map

Apart from embeddings, attention maps in the Transformers in ESM are also a popular feature in various downstream works. In essence, attention maps are cosine similarities between residues, capturing the correlation between different positions in a protein sequence.

An attention map can be directly used to predict the contact map because of their similar format [110, 111]. Additionally, ESM‐MSA’s attention map contains information of MSAs and is often utilized as an extra feature for predicting residue contacts [112]. Furthermore, paired MSAs are used for predicting residue–residue contacts in protein complexes [113, 114].

### 3.6 Used for evaluation and validation

ESM is also used for evaluation or validation within the workflows of various methods. There are two main categories here. Embeddings from models such as ESM‐1b, ESM‐1v, ESM‐MSA‐1b, and ESM‐2 are exploited to assist directed evolution [115, 116]. For example, Hu et al. [117] combined ESM‐1v’s embedding with their Bayesian optimization‐guided evolutionary algorithms and robotic experiments. ESM’s embeddings can also be used in the recycling of inverse folding as in CarbonDesign [118]. Another group uses ESMFold’s output for evaluation of protein structure, both in structure prediction tasks [119] and validation of mutation [120], where ESMFold generates structures for the proposed protein sequence with mutations in each iteration, which are used to calculate the energy function to guide the optimization process through simulated annealing.

As previously noted, the general language models underlying the ESM family are trained using MLM, making their outputs the probabilities of each amino acid at every position in the sequence. Consequently, leveraging these probability scores is a natural approach and has been extensively applied in downstream tasks, particularly in mutation effect prediction and sequence optimization, where labels can be predicted by position scores under many circumstances [70]. Apart from calculating the log‐likelihood ratio scores for mutation effect prediction [121, 122], probability distribution is also a source of features in the whole pipeline [123, 124] or the basis of evaluation [55]. Beyond individual position scores, researchers frequently compute sequence scores, which are functions of position scores in the sequence, mostly for evaluation [54, 125, 126]. ESM‐IF1 is frequently utilized for both scoring mutated sequences [92, 127] and computing self‐consistency perplexity to evaluate the generated protein structures [128]. Similarly, USCF ChimeraX [38], a widely used structure visualization tool, has recently started to employ ESMFold’s predicted local distance difference test score and predicted aligned error [17] to assess the accuracy of predicted structures.

## 4 DOWNSTREAM BIOLOGICAL APPLICATIONS

The ESM models, through large‐scale pretraining, capture the implicit structural and evolutionary information of protein sequences, thereby playing a role in multiple downstream tasks. These models are capable of implicitly learning how protein sequences determine their structures and functions without relying on explicit homology information. ESM models utilize techniques such as self‐attention mechanisms, contrastive loss functions, and Transformer architectures to identify key features of protein sequences, including secondary structures and long‐range interactions. They learn the characteristics of proteins by predicting masked amino acids and are able to enhance the predictive accuracy of specific tasks. Additionally, ESM models can be used for additional tasks, such as recommending evolutionarily plausible mutations, predicting the impact of mutations, and generating task‐specific molecular fingerprints in conjunction with graph neural networks. Although most papers we surveyed do not explain the specific working mechanisms of ESM models at the algorithmic level, they generally demonstrate the effectiveness and potential of ESM models in protein‐related tasks.

The availability of diverse datasets has further expanded the border of what is achievable with ESM. It can be seen that ESM models can perform many tasks provided a solid dataset exists. By surveying the papers we have collected, we find that ESM models have widespread applications in most popular fields of protein research.

### 4.1 Diverse tasks

Overall, various protein tasks in the downstream applications of ESM as well as their representative works are shown in Table 2. We will elaborate on each of them.

**TABLE 2:** Downstream applications of evolutionary scale modeling (ESM).

Field	Task	Applicable ESM models	Main application scenarios	Representative works (and key contributions)
Structure prediction	Fold	ESM‐1b, ESM‐MSA‐1b, ESM‐2	Classification of proteins based on structural similarities	Villegas‐Morcillo et al. [61] show that protein‐LM embeddings are beneficial for protein fold‐related tasks.
	Secondary structure		Prediction of secondary structure types and features	NetSurfP‐3.0 [57] achieves SOTA performance with significantly faster runtime on secondary structure (feature) predictions.
	Contact map		Distance between residues and 3D structure prediction	Rao et al. [110] reach SOTA unsupervised contact prediction performance with an end‐to‐end model.
	3D structure	ESM‐1b, ESM‐MSA‐1b, ESM‐2, ESMFold	Similar to the goal of the task and can be extended to the structure of any specific types of proteins, changes of protein conformations, etc.	OmegaFold [129]: The first method that predicts high‐resolution protein structures solely from a primary sequence, achieving AlphaFold2‐level accuracy on new targets and reliably modeling orphan proteins and antibodies.
	Protein complexes’ structure			Seq2Symm [130] predicts homo‐oligomer symmetry with SOTA performance, showing promise to be used together with structure generation methods.
Function prediction	EC	ESM‐1b, ESM‐MSA‐1b, ESM‐2	Prediction of protein’s catalytic behavior in biochemical reactions	SaProt [34] and ESM‐GearNet [35] explicitly fuse sequence and structural information and achieve improvements over existing sequence‐ and structure‐based models.
	GO		Prediction of biological process, molecular function, and cellular component	
	Text‐defined function		Prediction of general protein functions	ProtST [131] enhances protein sequence pretraining and understanding by biomedical texts; achieves significant performance in supervised learning, zero‐shot prediction, and functional protein retrieval.
	Functional annotation			NetGO 3.0 [132] significantly improves the performance of automated function prediction by leveraging information from unannotated proteins.
	Localization		Same as the name of the task	ProtGPS [133]: A PLM that predicts the compartment localization of human proteins with high performance; guides the generation of novel protein sequences that selectively assemble in the nucleolus; identifies pathological mutations that lead to altered subcellular localization of proteins.
	Solubility			NetSolP [33] predicts solubility and usability for purification of proteins expressed in *Escherichia coli* directly from the sequence, achieving SOTA performance and improving extrapolation across datasets.
	Homology detection			PLMSearch [134] is able to search millions of query‐target protein pairs in seconds while increasing the sensitivity and can recall remote homology pairs with dissimilar sequences but similar structures.
Interaction prediction	PPI		Prediction of binding (affinity) and residue contacts between proteins (including within homologous polymers)	DeepInter [113] predicts the inter‐protein residue‐residue contacts of protein complexes with SOTA performance.
	PLI		Prediction of protein‐ligand binding (affinity), antigen‐antibody interaction, antibody design, enzyme‐substrate interaction, and protein‐metal ion binding	DSMBind [63] proposes an unsupervised binding energy prediction framework that does not need experimental training data and discovers a novel PD‐L1 binder.
	DTI		Drug design	UdanDTI [135] outperforms SOTA models under in‐domain, cross‐domain, and structural interpretability settings of DTI; exceptionally accurate in predicting drug responses of two crucial subgroups of EGFR mutations associated with non‐small‐cell lung cancer.
Variant effect prediction		ESM‐1b, ESM‐1v, ESM‐IF1, ESM‐2	Prediction of functional properties of mutated sequences and the changes of functions between mutated and wild‐type sequences	ProteinGym [136] constructs a large‐scale and holistic set of benchmarks specifically designed for protein fitness prediction and design; devises a robust evaluation framework; reports the performance of models from various subfields into a unified benchmark suite.
Directed evolution			Antibody optimization, enzyme engineering, and protein sequence design for a given structure	EvoPlay [126]: A self‐play reinforcement learning framework to promote efficient sampling in the vast protein design space; supports AlphaFold2 as a structural surrogate to design peptide binders with high affinities; discovers variants with 7.8‐fold bioluminescence improvement beyond wild type.
Protein design	Unconditional generation	ESM‐IF1, ESM3	De novo protein design	Verkuil et al. [111] show that language models generalize beyond natural proteins to generate de novo proteins.
	Conditional generation		Designing proteins with desired functions or under given conditions (e.g., inverse folding), used in scenarios including sequence optimization, antibodies’ CDR design, and ligand‐binding protein design.	AntiFold [83]: SOTA inverse folding method on sequence recovery across CDR regions, with designed sequences showing high structural similarity to their solved counterparts.

#### 4.1.1 Structure prediction

Protein structure is categorized into four levels, from primary to quaternary. Aside from the primary structure, which is the amino acid sequence, the other structural levels can serve as targets for structure prediction. ESM can be effective at all these levels.

Firstly, secondary structure prediction typically involves classifying local structure [28, 32, 68]. Similar to this, there are predictions of certain structural properties, such as NetSurfP‐3.0 [57] using ESM‐1b to predict solvent accessibility, structural disorder, and backbone dihedral angles. Additionally, predicting intrinsically disordered regions is also a common task [53, 89].

The contact map represents the spatial proximity between residues in a protein structure, typically indicating whether two residues are within a certain distance threshold. It is a powerful tool for understanding protein folding and interactions, as well as guiding structure prediction. It is considered a feature between the secondary and tertiary structures of proteins, often used as a prediction target and as input for more complex protein structure or property prediction networks. The ESM team gives a good example directly using ESM‐1b to predict the contact map [110]. Additionally, MSA features are frequently explored in this task, as mentioned in Section 3.5.

A relatively straightforward problem in tertiary structure is fold classification. Protein families are generally categorized based on structural similarity, making this task a form of protein family classification. Classic datasets for fold classification primarily include Fold [137] and SCOPe, which have been utilized in many works [28, 61, 109, 138].

Protein structure prediction, which is defined as taking the protein sequence as input and predicting the coordinates of each atom in the three‐dimensional space (including tertiary and quaternary structures), is one of the most important topics in the field of protein, and the area is one of the most independent and popular subfields in protein research.

OmegaFold [139] uses ESM‐1b architecture to construct OmegaPLM, which is the key component of the ultimate structure prediction model. ModelAngelo [84] uses embeddings from ESM‐1b as inputs to GNN for automated analysis of cryo‐EMs. ESMPair [140] employs column attention from ESM‐MSA as one of the features to perform structure prediction of protein complexes. These all implicitly show that PLMs can act as qualified feature extractors, providing useful sequence information in diverse structure prediction tasks.

With the advent of remarkable models such as AlphaFold2 and ESMFold, the field has progressed rapidly to accurate prediction of protein three‐dimensional structures from primary sequences alone. Methods based on PLMs, including ESMFold, RGN2 [129], trRosettaX‐Single [141], OmegaFold [139], and HelixFold‐Single [142], are generally believed to leverage the evolutionary information implicitly learned by PLMs (such as ESM‐2) as a substitute for MSAs, positioning themselves as strong contenders against AlphaFold2, as shown in Table 3. A comparison of the network architectures, training datasets, and important hyperparameters of these PLM‐based methods can be seen in Ref. [39]. The main advantage of these PLM‐based methods over MSA‐based methods (such as AlphaFold2 and RoseTTAFold [143]) is fast prediction. Among them, ESMFold is typically considered to have the best overall performance and is good at tackling de novo designed proteins [144]. Meanwhile, trRosettaX‐Single shows excellent performance on orphan proteins [39], OmegaFold is suitable for antibodies [139, 145], and IgFold [130] is specifically designed for antibody structure prediction.

**TABLE 3:** Comparison between evolutionary scale modeling (ESM) and AlphaFold2.

Feature	AlphaFold2	ESM (e.g., ESMFold)
Core focus	Structure prediction	Sequence, structure, and functional prediction
Accuracy	Extremely accurate for structure prediction	Accurate but slightly less precise for structures
Speed	Slower due to reliance on MSAs	Faster, does not require MSAs
Applications	Structural biology, drug discovery, protein modeling	Synthetic biology, sequence design, evolution simulation
Scalability	Limited by computational cost for MSAs and training	Highly scalable for large datasets and generative tasks

Many studies have used ESMFold for domain‐specific protein structure predictions, thoroughly exploring its ability. The authors of ESMFold exploit it to perform large‐scale structural characterization of metagenomic sequences (ESM Metagenomic Atlas) [5]. Others explore multistate conformations of proteins [37, 64], attempt to understand the emergence and structural characteristics of de novo and random proteins [40], or predict protein homo‐oligomer symmetry by ESM‐2 [146]. USCF ChimeraX [38] not only uses ESMFold as a computational tool but also employs it to predict the various domains of the insulin receptor, fitting these domains to cryo‐EM maps to construct an initial model.

#### 4.1.2 Function prediction

Function prediction contains a wide range of tasks reflecting different aspects of protein functions. They are frequently adopted as prediction tasks where ESM models show their power.

Enzyme commission (EC) number prediction seeks to predict the EC numbers of different proteins, which describe their catalysis of biochemical reactions [147, 148]. Many works [34, 35, 78, 98, 105, 109] take EC number prediction as one of the tasks, and, surprisingly, all of them fuse outputs from ESM sequence models with structural information through different techniques. This shows that for structure‐related tasks, sequence information is not enough, and multimodality has become the trend. Gene ontology (GO) term prediction aims to predict whether a protein belongs to some GO terms, which classify proteins into hierarchically related functional classes [148, 149], and ESM models are popular models performing the task [29, 34, 35, 42, 78, 96, 105, 109]. The tasks of predicting EC and GO are both formulated as multilabel binary classification tasks and are the most widely used tasks in protein representation learning. Functional annotation prediction [32, 94, 132], homology detection [134], and solubility prediction [33, 94, 96] are also fields in which ESM models excel.

PLMs provide key spatial dimension information for systems biology through efficient subcellular localization prediction [34, 45, 56, 68, 94, 96, 133], which may potentially enhance the construction of biological networks, multiomics integration, and dynamic process simulation capabilities. In one of the latest works called ProtGPS [133], researchers propose that protein sequences contain a previously unrecognized code that controls their distribution in different subcellular compartments. Based on ESM‐2, they generate novel protein sequences that selectively assemble in the nucleolus and identify pathological mutations that lead to altered subcellular localization of proteins. The effort in the field has significant potential for application in the fields of disease mechanism analysis, drug development, and synthetic biology and is expected to become an important tool for systems biology research.

A special type of functional prediction involves text‐defined functions, and it perfectly matches the advantages of language models such as ESM. Traditional protein function prediction methods typically employ classification approaches that assign predefined labels based on protein characteristics. However, this approach often oversimplifies the complexity of protein functions, limiting our deep understanding of them. To address this limitation, some studies [67, 131] have proposed a new perspective that redefines protein functions using free‐text descriptions. Compared to traditional classification tasks, the range of labels for text‐defined functions is broader and more actionable. The CLIP framework is well‐suited for this task. For example, ProtST [131] pretrains a PLM alongside a biomedical language model (BLM) and a fusion module to jointly model protein sequences and biomedical texts. The paired PLM and BLM enable zero‐shot protein classification with only label descriptions, as well as retrieving functional proteins from a large database without requiring function annotations.

#### 4.1.3 Interaction prediction

Proteins exert their functions through interactions with other biomolecules, making the prediction of protein interactions crucial for understanding the roles of proteins in structural biology and drug discovery. In this field, ESM models represent a paradigm different from traditional docking models or structure prediction models such as AlphaFold2. Generally, ESM models generate more precise protein features to improve prediction performance instead of directly computing docking positions.

Interaction between individual proteins (i.e., protein‐protein interaction, or PPI for short) encompass a rich array of scenarios, such as the formation of protein oligomers, which are vital for studying the local features and functions of proteins. PPI tasks can be divided into protein level and residue level. At the protein level, ESM models are universally adopted as proteins’ global encoders both on binary classification to predict whether two proteins interact [28, 34] and on regression of the affinity or binding energy between proteins [28, 29, 63]. The residue‐level task focuses on predicting residue contact within PPI, determining which amino acids from the two interacting proteins come into proximity (i.e., with a distance smaller than a certain threshold) [91, 95, 112, 113, 114, 150]. Among these works, homologous oligomers are a focal point of interest [112, 114], where researchers take attention maps or MSA features into consideration as mentioned in Section 3.5, because they reflect interactions between different residues within or between proteins.

Proteins can also interact with other types of biomolecules, particularly small molecules, collectively referred to as ligands, to perform their diverse functions. Similar to PPIs, the application of ESM models in protein‐ligand interaction (PLI) involves predictions on whether binding occurs [34], the binding energy/affinity [28, 62, 63], and the identification of binding sites [96, 151]. These can be used in practical scenarios including antigen‐antibody interaction [62], antibody design [63], enzyme‐substrate interaction, and protein‐metal ion binding [34].

Furthermore, proteins can bind to drug molecules, enabling disease treatment. The task of predicting drug‐target interaction (DTI), which predicts whether a drug and a protein will bind, represents a highly active field due to its significance for drug discovery. ESM has already found some applications in this area [29, 99, 135] by offering high‐quality protein representations and holds significant promise for future developments.

#### 4.1.4 Variant effect prediction (VEP) and directed evolution

Variant effect, also referred to as mutation effect, generally means the functional properties of mutated sequences [136] or the change of functions between mutated sequences and wild‐type sequences, such as the change of Gibbs free energy [59, 60]. It has been shown that ESM models predict the effects of mutations by learning evolutionary patterns of protein sequences, which involve complex modeling of high‐dimensional relationships [66]. ESM models do not require explicit homology information and can estimate the probability for any possible amino acid sequence, which makes them suitable for the prediction of coding variants [121].

ESM models are frequently utilized for benchmarking [30, 31, 32, 136] or as base models in these VEP tasks, with representative datasets such as ProteinGym [136] (used in Refs. [34, 81, 87, 123, 152]), ClinVar [153] (used in ProteinGym’s works and Refs. [51, 121]), FLIP [31] (used in Refs. [68]), deep mutational scan data [30, 88, 92, 109], and those in EVmutation [154] (used in Refs. [32, 66]). Zero‐shot prediction of mutation effect [32, 34] is a popular topic and shows the transferability of ESM models. Domain‐specific problems [51, 55] also attract much attention. We notice that under many circumstances, raw ESM models were not SOTA [31] or could not satisfy performance requirements, and most researchers add other network modules [59, 60, 152], fuse information of other modalities [34, 35, 51, 87, 88, 109], or fine‐tune original models [66, 68, 70, 81, 92], just as the approaches to protein function prediction. Although the final hidden layer of the ESM model has been optimized for the unsupervised pretraining objective (learning to reproduce the masked sequence parts), this optimization may not be ideal for all the downstream tasks [68]. Techniques such as fine‐tuning enable the models to be better adapted to specific tasks, and additional information may be extracted directly from task‐specific training. Moreover, the scores computed by ESM models are sometimes used as training data [92].

Directed evolution, or sequence optimization, refers to the process of refining the amino acid sequence of a protein (i.e., continuously introducing mutations into the sequence) to enhance its performance or functionality, which imitates the process of natural selection in the laboratory. This strategy has been used in antibody optimization [122, 127] and enzyme engineering [117, 126]. It is also an approach to protein design [108]. ESM has been used in all of the above fields. As mentioned before, ESM models can be used to validate the sequences generated during the evolution process [115, 116, 124], thereby playing key roles in the directed evolution of proteins.

#### 4.1.5 Protein design

Protein design refers to the process of creating new proteins or modifying existing ones to achieve specific functions or properties. Designed proteins can be used in drug development, synthetic biology, vaccine production, and other biotechnological fields. Advancements in computational methods, such as those involving deep learning and generative models, have significantly enhanced the capabilities in protein design, enabling more complex and efficient designs. Here, we focus on protein design involving ESM.

Protein design can be generally divided into constrained generation and unconstrained generation. Unconstrained generation refers to the process of creating protein sequences or structures without strict limitations or predefined criteria [36, 111]. This approach allows for greater exploration of the sequence space, enabling the discovery of novel proteins with potentially unique properties or functions.

In contrast, constrained generation focuses on guiding the design process to ensure that the generated proteins meet certain functional, structural, or stability requirements. In constrained generation, inverse folding is a common task where a protein sequence is designed based on given coordinates of a protein backbone. ESM‐IF1 is a representative work in this area, which is a hybrid model consisting of a GVP‐GNN [16] structural encoder followed by a generic Transformer. Other ESM works in this area mainly use ESM models as components or validators. ProLLaMA [36] utilizes ESM2 as a component for unconditional and controllable protein sequence generation. EvoDiff [128] aims to generate proteins with intrinsically disordered regions and design scaffolds for functional structural motifs, employing ESM‐IF for scoring and evaluation. One of the later works by the ESM team [120] focuses on the programmatic design of sequences and structures, using ESMFold to assess the designed proteins. Apart from general protein design, designing for specific applications and targeting particular proteins holds significant promise for future advancements [63, 80, 83, 93, 102, 103, 125], and we will introduce these works in Section 4.2.

Unlike previous works, ESM3 achieves a joint design in terms of sequence, structure, and function. As long as any condition from these three aspects is input, an output can theoretically be obtained. This breaks the boundaries of traditional protein design and can be said to revolutionize the approach to protein design, making it adaptable to any scenario. However, at the time of writing this paper, there have been few follow‐up studies using ESM3, so its potential remains to be explored.

### 4.2 Various protein types

In addition to categorizing ESM’s downstream work based on task nature, the types of proteins present an interesting perspective. As mentioned earlier, as long as a dataset exists, ESM can be trained and tested on downstream tasks for any specific protein and its related tasks, providing convenience for biologists. From the existing downstream work on ESM, the diversity of protein types is evident, with several major categories represented.

Antibodies, due to their crucial role in the immune system and their close association with cancer immunotherapy, remain one of the most prominent subfields of protein research. The tasks related to antibodies can be generally classified into two categories. The first one is predicting antigen‐antibody binding, which can be viewed as a specialized and challenging PLI task. Such works include B‐cell epitope (the residues on the antibody that interact with the antigen) prediction [52, 58, 69, 86, 104] and antibody‐ligand binding energy prediction [62]. The second category involves the need to improve or design antibodies specifically targeted against certain antigens in clinical settings. Antibody affinity maturation [122, 127] refers to the process by which B cells undergo mutations in their antibody genes, leading to the production of antibodies with increased binding affinity for their specific antigens. This process is a key component of the adaptive immune response and occurs primarily in germinal centers of lymph nodes during an immune response. The design of antibody CDR (complementarity‐determining region) targeting specific antigens [63, 93] involves engineering the CDR sequences of antibodies to enhance their binding affinity and specificity. CDRs are the hypervariable regions of antibodies that interact directly with antigens, making them critical for determining an antibody’s effectiveness. Other works design antibody structure, particularly how to introduce sequence mutations without disrupting the antibody structure or its binding mode with the antigen [83].

In addition to antibodies, peptides are the most universally studied type of protein. Most of these works are aimed at peptide classification, especially signal peptides [47, 49, 77]. Signal peptides are also used as “prompts” in some other works [45, 46]. Other tasks include peptide screening [48], peptide property prediction [50], and peptide design in various practical scenarios [102, 103].

Enzyme engineering is an important field where the application of ESM is also significant. For instance, ESP [79] predicts small molecule substrates, Hu et al. [117] improve enzyme specificity (a class in directed evolution), and ESM‐Ezy [155] is able to identify superior enzymes with enhanced catalytic properties, especially for low‐similarity sequences. Johnson et al. [125] focus on the generation and function prediction of enzymes and enhance the experimental success rate of protein sequences generated from computational models.

There are many other types of proteins worth surveying, such as transmembrane proteins [46, 114] and fusion oncoproteins [80, 82]. However, due to the limited number of articles selected for this review, we will not elaborate on each one here. We encourage researchers to focus on biological questions and to establish more high‐quality data and tasks.

## 5 DISCUSSION

From this survey, it is evident that ESM models have demonstrated remarkable generalizability and transferability across different protein families and tasks, showcasing their potential for broad application in biological research and biotechnology.

However, it should be highlighted that ESM models are not omnipotent; that is, they do not perform well in all tasks. In antibody thermostability prediction [70] and protein sequence pretraining for structure prediction, zero‐shot mutation effect prediction, and out‐of‐domain generalization [156], for instance, traditional deep learning networks, including CNNs, show superior performance. Data bias is also a challenge. For example, the proteins used for pretraining most ESM models, that is, UniProt [14] sequences, are highly imbalanced across species. Specifically, although UniProt covers a wide range of organisms, the proteins from humans and several model organisms are significantly more abundant than those from viruses and some other mammals, and it is therefore difficult to apply ESM to studies on the latter species. Similarly, the numbers of proteins belonging to different categories or with different functions are also highly imbalanced. De novo designed proteins are not used in the pretraining of ESM models and are continuously updated. As such, although ESM predictions have shown good quality on proteins with keywords “de novo” or “designed” in PDB [39], their applications on newly designed proteins require further validation. In short, current pretraining data may not represent the real distribution of the whole protein space, and the shift between the distributions of downstream task‐specific data and pretraining data can affect the performance of ESM. Therefore, the applications of ESM in specific scenarios require an understanding of the data.

Another challenge ESM may encounter in practical scenarios is high computational resource demands. Specifically, the time complexity of Transformer‐based models is typically considered *O*(*n* ^2^ *d*), where *n* is the sequence length and *d* is the hidden dimension of the input [13]. In addition, memory consumption increases drastically with the scaling of model size, especially when some of the parameters are unfrozen. For example, a model with 1B parameters trained with the Adam optimizer and single‐precision floating‐point consumes at least 16G memory. Consequently, although ESM models are often able to make fast inferences on a single sequence, they are still costly in terms of running time and memory usage when the sequence is very long, there are large amounts of data, or the model needs to be fine‐tuned or retrained, especially for those models with large numbers of parameters (e.g., ESM‐2‐15B and ESM3‐98B.). The lack of model scalability could constrain the range of ESM’s practical applications.

Although the predictive power of ESM is well‐established, the interpretability of these models remains an area of active exploration. Efforts to demystify the “black box” of deep learning are ongoing, with approaches such as attention visualization and gradient analysis providing insights into the model’s decision‐making processes. For instance, Zhang et al. [157] show that ESM‐2 stores statistical information about coevolving residues by calculating the “categorical Jacobian.” Valeriani et al. [138] analyze the intrinsic dimension of data representation in the ESM‐2 model and the variations in neighbor composition, as well as how these changes occur between different layers of the model. Nevertheless, these findings are still a long way from in‐depth interpretability, which involves clarifying how ESM “learns” biological knowledge and functions in downstream biological tasks.

In conclusion, the ESM series has significantly advanced the field of AI for protein science and engineering, offering a powerful toolkit for researchers and clinicians alike. As related technology matures, we anticipate more fruitful integration of ESM into biological research, leading to new discoveries and innovations in pharmacology and biotechnology. The future of ESM looks promising with ongoing developments aimed at enhancing model interpretability, improving coordination with experimental data, and expanding the range of applications in more areas of life sciences and bioengineering, such as synthetic biology and systems biology.

## AUTHOR CONTRIBUTIONS

**Qingyu Yang**: Formal analysis; investigation; visualization; writing—original draft. **Jiale Yu**: Visualization; writing—review and editing. **Jie Zheng**: Supervision; writing—review and editing.

## CONFLICT OF INTEREST STATEMENT

The authors declare no conflicts of interest.

## ETHICS STATEMENT

This review article does not involve any research related to human or animal subjects.

## Data Availability

Data sharing is not applicable to this article as no datasets were generated or analyzed during this study.
